# Role of ACTH in the Interactive/Paracrine Regulation of Adrenal Steroid Secretion in Physiological and Pathophysiological Conditions

**DOI:** 10.3389/fendo.2016.00098

**Published:** 2016-07-20

**Authors:** Hervé Lefebvre, Michaël Thomas, Céline Duparc, Jérôme Bertherat, Estelle Louiset

**Affiliations:** ^1^U982, Laboratory of Neuronal and Neuroendocrine Differentiation and Communication, INSERM, Institute for Research and Innovation in Biomedicine, Mont-Saint-Aignan, France; ^2^Normandie Université, UNIROUEN, Rouen, France; ^3^Department of Endocrinology, Diabetes and Metabolic Diseases, University Hospital of Rouen, Rouen, France; ^4^U1016, INSERM, Institut Cochin, Paris, France; ^5^Department of Endocrinology and Metabolic Diseases, Hôpital Cochin, Assistance Publique-Hôpitaux de Paris, Paris, France

**Keywords:** ACTH, aldosterone, cortisol, Cushing’s syndrome, aldosterone-producing adenoma, hyperplasia, adrenocortical cells, VEGF

## Abstract

In the normal human adrenal gland, steroid secretion is regulated by a complex network of autocrine/paracrine interactions involving bioactive signals released by endothelial cells, nerve terminals, chromaffin cells, immunocompetent cells, and adrenocortical cells themselves. ACTH can be locally produced by medullary chromaffin cells and is, therefore, a major mediator of the corticomedullary functional interplay. Plasma ACTH also triggers the release of angiogenic and vasoactive agents from adrenocortical cells and adrenal mast cells and, thus, indirectly regulates steroid production through modulation of the adrenal blood flow. Adrenocortical neoplasms associated with steroid hypersecretion exhibit molecular and cellular defects that tend to reinforce the influence of paracrine regulatory loops on corticosteroidogenesis. Especially, ACTH has been found to be abnormally synthesized in bilateral macronodular adrenal hyperplasia responsible for hypercortisolism. In these tissues, ACTH is detected in a subpopulation of adrenocortical cells that express gonadal markers. This observation suggests that ectopic production of ACTH may result from impaired embryogenesis leading to abnormal maturation of the adrenogonadal primordium. Globally, the current literature indicates that ACTH is a major player in the autocrine/paracrine processes occurring in the adrenal gland in both physiological and pathological conditions.

## Introduction

The adrenal cortex is a heterogeneous tissue that not only contains steroidogenic cells but also hosts various cell types that are able to locally release a wide variety of bioactive signals. This histological organization results in a complex interactive network that participates in the regulation of both basal and ACTH-induced corticosteroidogenesis. The intracortical sources of regulatory factors include chromaffin cells arranged in cords or islets, nerve fibers originating from extraadrenal neurones or cell bodies located in the adrenal medulla, cells of the immune system, including lymphocytes, macrophages/monocytes and mast cells, endothelial cells, and adipocytes. These autocrine/paracrine mechanisms have been extensively reviewed during the past years (1–5). However, their physiological role, especially their exact contribution to the regulation of corticosteroid synthesis remains a matter of debate although some data indicate that intraadrenal regulatory signals may mediate parts of the biological effects of ACTH on the adrenal cortex. Interestingly, adrenocortical neoplasms associated with steroid hypersecretion exhibit molecular and cellular defects that tend to reinforce the potency of paracrine factors to activate corticosteroidogenesis. For instance, abnormal expression of ACTH has been reported in adrenocortical cells in both tumors and hyperplasias responsible for hypercortisolism (6–11).

In the present review article, we have updated the current knowledge on the role of ACTH in the cell-to-cell communication processes occurring in the adrenal cortex in both physiological and pathological conditions. We will also discuss the potential interest of the intraadrenal ACTH regulatory loop for the clinical management of patients with primary adrenal excess of corticosteroids.

## Effect of ACTH on the Adrenal Vasculature

**Figure 1:**
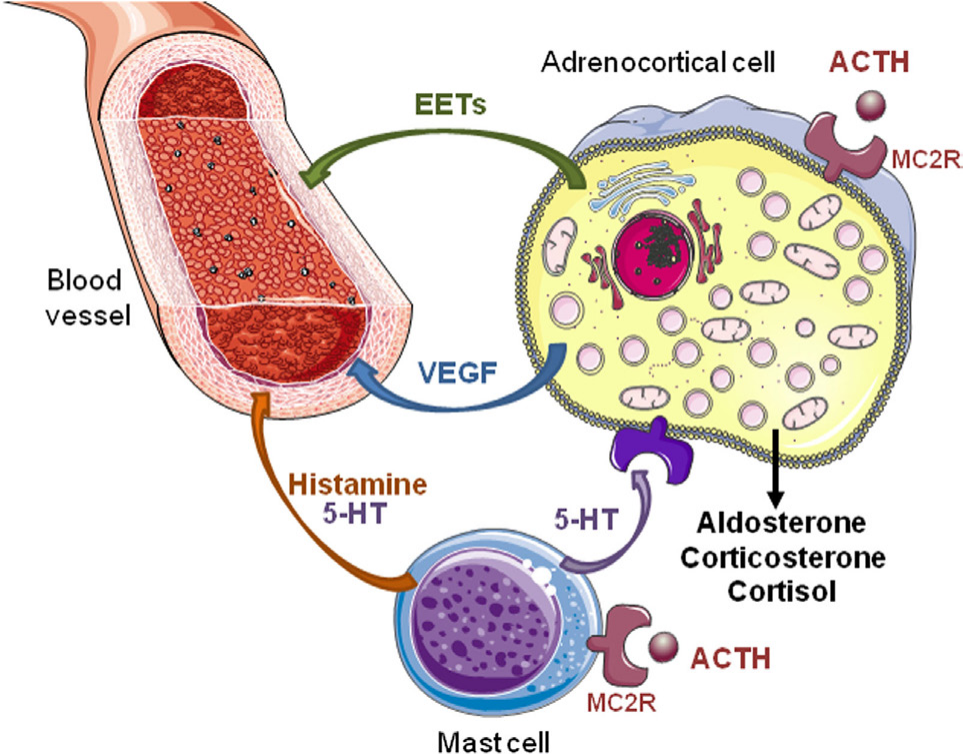
**Direct and indirect effects of ACTH on steroidogenesis in the adrenal gland**. ACTH exerts a direct stimulatory effect on adrenocortical cells *via* melanocortin type 2 receptors (MC2R). Activation of MC2R increases secretion of steroid hormones, vascular endothelial growth factor (VEGF), and epoxyeicosatrienoic acids (EETs). Angiogenesis induced by VEGF, an endothelial cell-specific mitogen factor, allows adrenal tissue growth in response to ACTH. Increase in adrenal blood flow elicited by EETs, which are potent vasorelaxant agents, favors steroid production. In rats, ACTH also activates adrenal mast cells inducing local release of histamine and serotonin (5-HT). These factors modulate the tonicity of adrenal arterioles and, thus, the adrenal blood flow that indirectly influences the steroidogenesis. In addition, 5-HT stimulates steroid secretion through activation of different 5-HT receptor subtypes expressed by normal and tumor human adrenocortical cells.

The adrenal cortex is a richly vascularized organ. This extensive vasculature is essential for delivery of tropic hormone and steroid hormones precursors to the gland and secretion of mature hormones into the blood flow. Furthermore, the establishment of such dense vascular network ensures that every adrenocortical cell is in contact with at least one endothelial cell (12, 13). This remarkable histological organization allows paracrine regulation of adrenocortical cells by endothelial cells through release of endothelins, adrenomedullin, nitric oxide, and prostacyclin (14). For instance, Rossi et al. have shown that endothelin-1 released by endothelial cells is an important regulator of aldosterone secretion, and may then indirectly influence arterial blood pressure (15, 16). The release of endothelin by adrenocortical sinusoids is thought to mediate the modulation of adrenal steroidogenesis by the adrenal blood (17). Interestingly, ACTH appears able to both act on development and maintenance of the adrenal vasculature and regulate the adrenal blood flow (18, 19), influencing thus steroid production through an indirect effect in addition to its intrinsic steroidogenic action on adrenocortical cells (Figure 1).

The modulation of adrenal angiogenesis by ACTH may involve several bioactive signals. Thrombospondins (TSPs) represent a wide family of extracellular proteins consisting of five members, TSP1–5, which can bind multiple cell surface molecules, including heparin sulfate proteoglycans, low-density lipoprotein receptor-related protein, integrins, CD36, and CD47 (20). Owing to the great diversity of their binding partners, TSPs are involved in various biological processes, such as cell adhesion, spreading and migration, and angiogenesis (20, 21). In this respect, TSPs are known to inhibit angiogenesis by preventing migration of capillary endothelial cells. Interestingly, adrenocortical cells release high amounts of TSP2 in response to ACTH (22), suggesting that TSP2 may mediate some of the biological actions of ACTH in the adrenal cortex, especially centripetal adrenocortical cell migration, which is a fundamental process in the dynamic organization and remodeling of the adrenal cortex (23). However, the physiological role of TSP2, which is primarily expressed in zona glomerulosa and zona fasciculata, remains unclear since TSP2-null mice exhibit no alteration in corticosteroid secretion or adrenal development (24). In addition, syndromes of ACTH excess, including Cushing’s disease and 21-hydroxylase deficiency, are associated with adrenocortical hyperplasia, a process that underlies active angiogenesis. Conversely, several observations indicate that vascular endothelial growth factor (VEGF) plays a pivotal role in the trophic effects of ACTH on the adrenal vasculature (25). VEGF is a widely expressed cytokine that acts as an endothelial cell-specific mitogen and angiogenic factor. In the bovine adrenal gland, VEGF is expressed in zona glomerulosa and zona fasciculata cells and its release is stimulated by ACTH (26). Upregulation of VEGF by ACTH has also been reported in human adrenal (27). The effect of ACTH on adrenal VEGF production involves transcription-independent mechanisms, including stabilization of VEGF mRNA by the HuR protein (28). Conversely, ACTH suppression by dexamethasone in mice results in progressive decrease of VEGF expression in adrenocortical cells and regression of the vascular network (29). Interestingly, ACTH also stimulates VEGF expression in human fetal adrenocortical cells, suggesting that VEGF is an important mediator of the trophic action of ACTH during the adrenal development (30). Consistently, an increase in VEGF expression in adrenocortical cells has been noticed in the regenerating adrenal cortex in rats (31, 32). It is noteworthy that, in parallel to its effect on adrenal vasculature development *via* local synthesis of VEGF, ACTH also favors adrenal tissue growth through an antiapoptotic action on adrenocortical cells (29) and stimulation of synthesis of growth factors (30). Finally, VEGF may be involved in the capacity of ACTH to induce endothelial fenestration, a phenomenon that favors cell-to-cell interactions (26, 33).

The mechanism by which ACTH modulates the adrenal blood flow is obviously not univocal. Adrenocortical cells themselves are able to release vasorelaxant agents in response to ACTH. These compounds include metabolites of arachidonic acid, such as epoxyeicosatrienoic acids (EETs) (34). In addition, in the rat adrenal gland, capsular mast cells modulate the tonicity of adrenal arterioles and, thus, the adrenal blood flow through local release of histamine and serotonin (5-hydroxytryptamine; 5-HT) (17, 35). Interestingly, rat adrenal mast cells are sensitive to the action of ACTH, suggesting that they may represent an important intermediate in the effect of corticotropin on the adrenal blood flow (35). In humans, the presence of mast cells has been reported in both the subcapsular region of the normal adrenal gland (36) and various types of adrenocortical tumors, including deoxycorticosterone-secreting tumors, aldosterone-producing adenomas (APAs), and adrenocortical carcinomas (37–39). However, there is currently no data reported in the literature indicating that human adrenal mast cells are target cells for ACTH.

## Indirect Effects of ACTH on Corticosteroidogenesis through Adrenocortical Cell Secretory Products

Adrenocortical cells are important sources of bioactive compounds that are able to modulate steroidogenesis through autocrine/paracrine actions. In man, it is doubtful that corticosteroid may affect their own secretion in physiological conditions. Conversely, the adrenal cortex is known to express the diverse components of the renin–angiotensin system, leading to local synthesis of angiotensin II and potential autocrine/paracrine stimulation of aldosterone secretion (40). Interestingly, renin production, which primarily occurs in zona glomerulosa cells, is stimulated by ACTH and reduced by hypophysectomy or dexamethasone administration (41, 42). This observation may explain why plasma renin levels are usually not suppressed in patients with overt ACTH-dependent hypercortisolism (43, 44). In fact, it is conceivable that, in this condition, ACTH-induced adrenal renin secretion may compensate inhibition of renal renin synthesis by hypervolemia and hypertension secondary to cortisol excess.

Adrenocortical cells also synthesize and release various cytokines, such as interleukin-1 (IL-1), IL-3, IL-6, and tumor necrosis factor-α (TNF- α) (1). These signals may influence the steroidogenic and mitogenic activities of adrenocortical cells *via* autocrine/paracrine processes (5). Indeed, ACTH has been shown to modulate cytokine production by adrenocortical cells, either positively (IL-6) or negatively (TNF-α) (45, 46). Intraadrenal cytokines may, thus, represent important effectors of ACTH capable of potentiating or attenuating its action on adrenocortical cells. However, there is currently no evidence that the impact of ACTH on adrenal cytokine synthesis may be involved in the pathogenesis of ACTH-dependent adrenal hyperplasia and/or hypercortisolism.

Several types of adrenocortical neoplasms are associated with illicit neuroendocrine differentiation of adrenocortical cells (47). This is especially true for APAs that have been shown to express synaptophysin, neuronal cell adhesion molecule, neuron specific enolase, and SV2 (48, 49). In addition, we have observed that APA cells may also abnormally synthesize 5-HT that is able to stimulate aldosterone secretion through activation of the overexpressed serotonergic type 4 (5-HT4) receptor (50, 51). Because APA cells also express high amounts of the ACTH receptor, i.e., the melanocortin receptor type 2 (MC2R) (51–53), it is conceivable that locally produced 5-HT may act as an amplifier of the stimulatory effect of ACTH on aldosterone secretion by APA tissues.

Inhibins and activin are dimeric peptides belonging to the TGF-β family. Inhibins are formed by combination of the α-subunit encoded by INHA and A or B isoform of the β-subunit, encoded by *INHBA* and *INHBB*, respectively. Alternatively, activin is a homodimer composed of two β-subunits. The action of activin is mediated by its specific receptors type I and II, and the intracellular proteins SMAD. Inhibins counteract the biological effects of activin by antagonizing activin type II receptor and formation of an inactive complex with the TGFβ type III receptor β-glycan. Adrenocortical cells are able to express both α and β subunits (54–56). In particular, the α-inhibin is expressed in the zona reticularis under the positive control of ACTH, whereas β-subunits are mainly present in the outer cortex. Both activin receptors and the inhibin co-receptor β-glycan are also detected in the adrenal cortex (55, 56). It has been demonstrated that ACTH stimulates secretion of inhibin A and B, without modifying production of activin A (55). These data indicate that ACTH also controls corticosteroidogenesis through modulation of the intraadrenal activins/inhibins ratio.

## Indirect Effects of ACTH on Corticosteroidogenesis through Non-Steroidogenic Adrenal Cells

The adrenal gland is surrounded by adipose tissue and its cortical region contains adipocytes either isolated or arranged in small islets (57). Like cells of the immune system, adipocytes release a wide panel of cytokines, suggesting that they could influence the adrenocortical function through a cell-to-cell communication process. For instance, adipocytes have been shown to activate aldosterone release by secreting soluble bioactive factors, which have not been yet characterized (57–59). Conversely, leptin exerts an inhibitory action on ACTH-induced corticosteroid secretion in human adrenocortical cells without affecting their viability and proliferation (60–63). It is possible that peri- and intraadrenal adipocytes may be controlled by ACTH and, thus, constitute a relay in the action of the hormone on the adrenal cortex. In support of this hypothesis, it has been reported that murine adipocyte cell lines and immortalized adipocytes express the MC2R, and ACTH regulate adipocyte functions in these models (64–66). In addition, patients with congenital adrenal hyperplasia due to 21-hydroxylase deficiency, a condition which is associated with chronically high plasma ACTH levels, can present with adrenal myelolipoma (67–69), suggesting a role of ACTH in the development of lipomatous tissue inclusions in the adrenal glands. However, in contrast to the observations made in murine cells, human mature adipocytes only express low levels of the MC2R and ACTH does not influence lipolysis in the mature human adipose tissue (70).

## Paracrine Control of Adrenal Steroidogenesis by Intraadrenal ACTH

**Figure 2:**
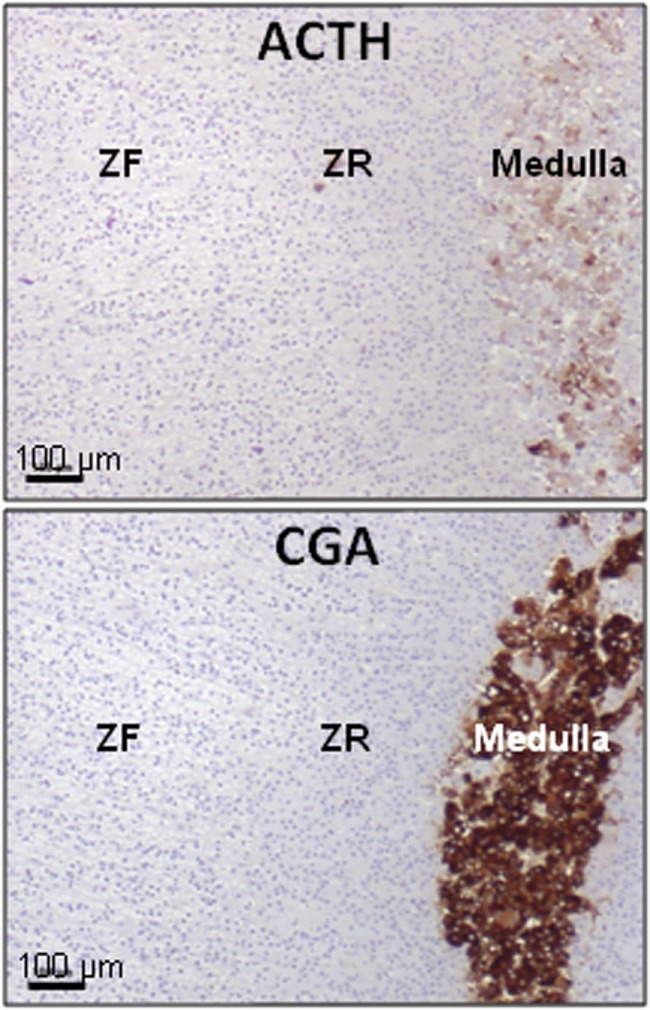
**Presence of ACTH in the normal human adrenal**. ACTH immunoreactivity is exclusively located in some chromaffin cells (high panel), identified as chromogranin A (CGA) immunoreactive cells (low panel), in the medulla. ACTH was detected by using antibodies against the N-terminal region of the peptide. ZF, zona fasciculata; ZR, zona reticularis. Illustration of the data published in Ref. (9).

In the normal adrenal gland, chromaffin cells release detectable amounts of ACTH that is a major mediator in the corticomedullary functional interaction (71, 72) (Figure 2). This secretory process can be activated by corticotropin-releasing hormone (CRH) that is expressed in the adrenal medullary tissue (11). As chromaffin cells are also regulated by splanchnic nerves and proinflammatory cytokines, it seems possible that they may be important intermediates in the activation of the adrenal cortex during stress and inflammation. However, the physiological role of the paracrine control of corticosteroidogenesis by adrenomedullary ACTH remains unclear (3).

Conversely, clinicopathological studies have shown that paracrine interactions involving ACTH produced by chromaffin cells may play a role in the pathogenesis of hypercortisolism. Pheochromocytoma can occasionally produce ACTH resulting in ACTH-dependent Cushing’s syndrome (73–75). Hypercortisolism seems to be mainly the consequence of the endocrine corticotropic action of ACTH whose plasma levels are typically elevated in this situation (76). Nevertheless, this mechanism may not be exclusive. In fact, hyperplasia of the adrenal cortex adjacent to pheochromocytoma has also been observed, indicating that ACTH originating from the pheochromocytoma tissue can stimulate adrenocortical cells in a paracrine manner (11, 77–80). Such a histological pattern is close to what is observed in corticomedullary mixed tumors that are composed of intermingled adrenocortical and pheochromocytoma tissues (81–84). These rare tumors are sometimes associated with hypercortisolism, suggesting that pheochromocytes release paracrine signals capable of activating glucocorticoid synthesis (81, 84). ACTH could be one of them although it is not excluded that catecholamines may exert a stimulatory action on cortisol production through illicit expression of adrenergic receptors in tumor adrenocortical cells (85, 86).

**Figure 3:**
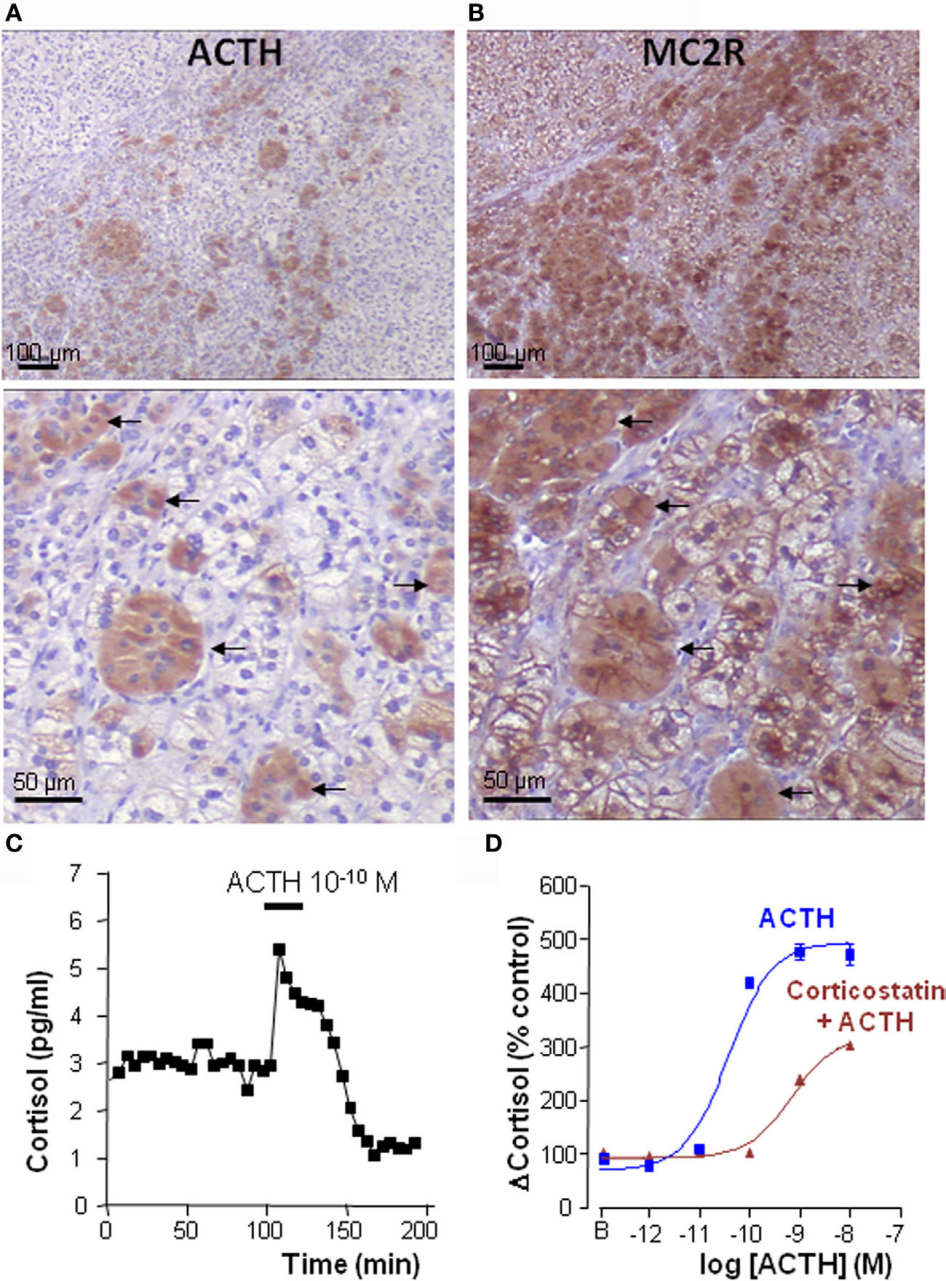
**Intraadrenal ACTH regulation of cortisol secretion in bilateral macronodular adrenal hyperplasia (BMAH)**. **(A,B)** Consecutive sections of a BMAH tissue labeled with ACTH and MC2R antibodies. **(A)** Heterogeneous distribution of ACTH-producing cell clusters in BMAH. **(B)** The ACTH receptor MC2R is highly expressed in ACTH-producing cells (arrows) and in their vicinity. **(C)** ACTH sensitivity of BMAH tissues. Application of exogenous ACTH stimulated cortisol secretion by perifused BMAH explants. **(D)** Corticostatin, a MC2R antagonist, inhibited *in vitro* the cortisol response of cultured BMAH cells to ACTH. Illustration of the data published in Ref. (9).

Alternatively, the occurrence of ectopic production of ACTH in the adrenal cortex has already been reported. Very rarely, ACTH-positive cells in the adrenocortical tissue can reveal adrenal micrometastases of an ACTH-secreting cancer. In the published cases, plasma ACTH levels were strongly elevated, as a result of ACTH secretion by the primary tumor (87–89). More surprisingly, a subpopulation of adrenocortical cells has been shown to produce detectable amounts of ACTH in various types of adrenal neoplasms. A first case of adrenocortical cortisol-secreting adenoma associated with production of ACTH by tumor cells, has been described in 2001 by Hiroi et al. (6). Illicit synthesis of ACTH was considered to result from abnormal pituitary differentiation of the tissue as witnessed by co-expression of 17-hydroxylase and pituitary homeobox factor-1 mRNAs by adrenocortical cells (6). At the ultrastructural level, tumor cells exhibited characteristics of both steroidogenic cells and neuroendocrine cells, and the tumor was, thus, referred to as an adrenocortical–pituitary hybrid adenoma. Bilateral macronodular adrenal hyperplasia (BMAH), a rare cause of primary adrenal hypercortisolism, has also been found to contain ACTH-producing cells. This observation was first reported by Pereira et al. who also noticed that ACTH-positive cells were labeled by antibodies to chromogranin A (CGA), suggesting that these cells may correspond to intracortical chromaffin cells (90). Subsequently, several teams reported expression of proopiomelanocortin (POMC) and ACTH in groups of adrenocortical cells in isolated BMAH cases (8, 10, 91) (Figure 3). In one case, it could be shown that ACTH-positive cells also expressed 17-hydroxylase but were negative for pituitary corticotroph markers (8). The role of ACTH in the pathogenesis of BMAH has been more extensively investigated in a large series of 30 cases (9). Adrenal hyperplasia samples expressed POMC mRNA at variable levels. Proconvertase 1, a protease implicated in the maturation of POMC into ACTH, was also visualized in clusters of adrenal cells, indicating that ACTH could be generated from POMC in the BMAH tissues. In agreement with this finding, ACTH immunoreactivity was detected, as previously noticed, in some adrenocortical cells either isolated or arranged in small groups disseminated in the tissues. The presence of corticotropin can also be seen in some chromaffin cells of the adrenal medulla. Surprisingly, adrenocortical ACTH-positive cells also displayed characteristics of steroidogenic cells, such as the presence of numerous lipid inclusions and several markers of steroidogenic differentiation, including steroidogenic factor 1 (SF1), 17-hydroxylase, and the HDL-cholesterol receptor scavenger receptor B1 (SRB1). It could be, thus, concluded that they constitute a subcategory of adrenocortical steroidogenic cells characterized by an unusual capacity to synthesize ACTH. BMAH specimens were found to express very low levels of T-pit [a transduction factor which controls pituitary corticotrophs differentiation (92)], confirming that ectopic synthesis of ACTH in adrenocortical cells does not result from illicit corticotropic-like differentiation of the latter (9) but may rather be considered as an additional feature of neuroendocrine differentiation of the hyperplastic tissues (47, 93, 94). Interestingly, ACTH-containing cells were also positive for gonadal markers like the gonadal marker insulin-like 3 (INSL3), exhibiting thus a pseudo-gonadal phenotype (8, 9, 95, 96). This observation is concordant with expression of POMC and synthesis of ACTH previously reported in testicular Leydig cells and ovarian granulosa cells (97, 98). Because the adrenal glands and gonads both originate from a same tissue precursor, the adrenogonadal primordium, it is likely that the occurrence of these pseudo-gonadal cells in BMAH tissues may be the consequence of altered embryogenesis, explaining the bilaterality of the lesions.

Perifusion studies revealed that ACTH is released by BMAH tissues *in vitro* in a pulsatile mode (9), in agreement with older clinical studies showing a clear pulsatility of cortisol secretion in patients with BMAH (99). The ectopic secretion of ACTH by the hyperplastic adrenal glands has also been detected *in vivo* in two patients through adrenal vein catheterization (9). Taken collectively, these data suggested that, in BMAH tissues, intraadrenal ACTH may exert an autocrine/paracrine action to stimulate cortisol secretion, supplying therefore circulating ACTH that is suppressed by cortisol excess. This hypothesis was supported by statistical analyses showing positive correlations between ACTH and cortisol levels in BMAH culture medium. In addition, basal plasma cortisol concentrations measured *in vivo* were positively correlated with both the levels of POMC mRNA and the ACTH histological score in the tissues. Most importantly, MC2R antagonists, such as corticostatin and ACTH (7–38), were found to inhibit *in vitro* spontaneous and ACTH-evoked cortisol secretion by BMAH explants (9) (Figure 3). Clinical studies with MC2R antagonists are now mandatory to confirm that cortisol production is actually dependent on intraadrenal ACTH in patients with BMAH.

Bilateral macronodular adrenal hyperplasia tissues also constitute an interesting model for the study of the regulation of MC2R expression in the adrenal cortex. MC2R mRNA is globally underexpressed in BMAH samples versus normal adrenals (100). In fact, we could observe that, at variance with the normal adrenal cortex that is diffusely labeled by anti-MC2R antibodies, BMAH explants exhibit heterogeneous distribution of the receptor that appears highly expressed in the vicinity of clusters of ACTH-producing cells and more weakly at distance (Figure 3). Indeed, as previously established in the normal adrenal gland (101–103), MC2R seems to be upregulated by ACTH in BMAH tissues. In fact, MC2R mRNA levels were positively correlated with POMC mRNA rates and MC2R-like immunoreactivity was principally visualized in the vicinity of ACTH-positive cells (9). Interestingly, ACTH-producing cells were also found to express the receptor, suggesting that intraadrenal ACTH possibly exerts autocrine actions in BMAH.

All these data indicate that intraadrenal ACTH plays a pivotal role in the pathogenesis of hypercortisolism associated with BMAH. Deciphering the mode of regulation of ACTH production by BMAHs is, thus, essential for the comprehension of the pathophysiology of the disease. At variance with pituitary ACTH, intraadrenal ACTH does not seem to be regulated by cortisol, as suggested by the lack of action of dexamethasone and the glucocorticoid receptor antagonist RU486 on ACTH release by BMAH explants (9). Conversely, we noticed that ligands of various membrane receptors that are known to be abnormally expressed by BMAH cells, i.e., 5-HT, LH/hCG, and glucose-dependent insulinotropic peptide (GIP), are able to activate ACTH production from BMAH tissues *in vitro* (9). This surprising finding indicated that activation of illicit membrane receptors may stimulate cortisol production *via* two mechanisms, including a direct effect on corticosteroidogenesis, as previously shown in BMAH cell culture (93, 104), and an indirect action mediated by ACTH secretion (9). Consistently, MC2R antagonists were found to partially inhibit *in vitro* the cortisol response evoked by GIP in perifused BMAH samples. Taken together, these results suggest that intraadrenal ACTH may be regarded as a common intermediate and amplifier of the action of several illicit membrane receptors in BMAH tissues. Targeting the MC2R with specific antagonists may, thus, represent an efficient strategy for the treatment of BMAH-associated hypercortisolism. The pathophysiology links between intraadrenal ACTH and abnormally expressed receptors may be more complex and could form a complete auto-amplification loop in the hyperplastic tissues. In fact, although the presence of the LH and GIP receptors in adrenocortical cells may be simply considered as features of pseudo-gonadal differentiation of BMAH tissues, overexpression of some membrane receptors, such as 5-HT receptors, may result from local production from ACTH. This hypothesis is supported by intriguing observations recently performed in another type of adrenal hyperplasia associated with hypercortisolism, namely primary pigmented adrenocortical disease (PPNAD). The disease is caused in most patients by germline inactivating mutations that affect the *PRKAR1A* gene (105), resulting in constitutive activation of protein kinase A (PKA) in adrenocortical cells (106). Since MC2R are positively coupled to the cAMP/PKA pathway, it can be considered that *PRKAR1A* mutations partly mimic the action of ACTH on adrenal steroidogenic cells. Interestingly, PPNAD have been found to overexpress several types of 5-HT receptors, including the 5-HT_4_ receptor (107). It seems, thus, conceivable that abnormal expression of 5-HT receptors in BMAH tissues may result from exposure of adrenocortical cells to locally produced ACTH whose release is subsequently increased by the 5-HT signaling pathway.

**Figure 4:**
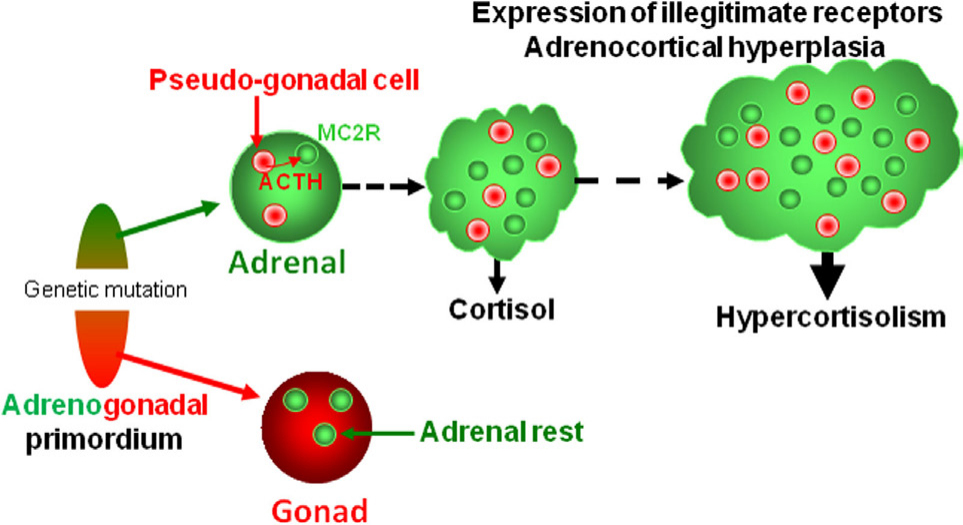
**Putative pathophysiological mechanism responsible for bilateral macronodular adrenal hyperplasia**. Both gonads and adrenals originate from the adrenogonadal primordium. It can be speculated that the causative genetic mutations may alter differentiation and/or separation of the adrenogonadal primordium leading to the presence of pseudo-gonadal cells in the adrenals. Secretion of ACTH by intraadrenal pseudo-gonadal cells may progressively stimulate, *via* activation of MC2R, both cortisol secretion and growth of the adrenocortical tissue, leading to bilateral adrenal hyperplasia associated with hypercortisolism. In parallel, sustained activation of the PKA pathway consecutive to activation of MC2R by intraadrenal ACTH may activate expression of some illegitimate receptors. Activation of steroidogenesis by ligands of illegitimate receptors further reinforces cortisol hypersecretion through both an intrinsic stimulatory action and an indirect effect *via* local release of ACTH.

There is now no doubt that BMAH is a genetically determined condition, *ARMC5* being a major susceptibility gene of the disease (108). It is now well established that the development of macronodular adrenal hyperplasia requires inactivation of the two *ARMC5* alleles, respectively, by the first germline mutation and a secondary somatic genetic event. Surprisingly, inactivation of *ARMC5* expression in the human adrenocortical cell line H295R, which reproduces the molecular defects observed in adrenocortical cells of patients with BMAH, results in a decrease in expression of steroidogenic enzymes (108, 109). It seems, thus, that a second line event is necessary for the emergence of hypercortisolism. We hypothesize that *ARMC5* mutations may alter differentiation and/or separation of the adrenogonadal primordium leading to the presence of pseudo-gonadal cells in the adrenal areas. Progressive expression of POMC and ACTH by these cells may then result in cortisol hypersecretion. Because illicit expression of ACTH has also been observed in non-ARMC5-mutated adrenal hyperplasias, it seems that abnormal differentiation of adrenocortical cells is a frequent histological feature in BMAH tissues whatever the causative firstline genetic defect (Figure 4).

## Conclusion

In addition to its well-known action on adrenocortical cells to promote steroidogenesis through activation of the MC2R, ACTH exerts multiple effects through the paracrine communication processes that occur in the adrenal gland in both physiological and pathophysiological conditions. ACTH can also be abnormally produced in adrenal neoplasms in which the hormone acts, thus, as an autocrine/paracrine factor to activate steroid secretion. This new pathophysiological concept opens novel avenues for the development of original pharmacological treatments of primary adrenal syndromes of steroid excess.

## Author Contributions

HL, MT, and EL: wrote the paper. CD, EL: acquisition, analysis, and interpretation of data. EL: designed the figures. JB: revised the paper and gave intellectual contribution.

## Conflict of Interest Statement

The authors declare that the research was conducted in the absence of any commercial or financial relationships that could be construed as a potential conflict of interest.
